# Humanin and Age-Related Diseases: A New Link?

**DOI:** 10.3389/fendo.2014.00210

**Published:** 2014-12-04

**Authors:** Zhenwei Gong, Emir Tas, Radhika Muzumdar

**Affiliations:** ^1^Department of Pediatrics, University of Pittsburgh School of Medicine, Pittsburgh, PA, USA; ^2^Department of Pediatrics, Division of Pediatric Endocrinology, Children’s Hospital of Pittsburgh of UPMC, Pittsburgh, PA, USA; ^3^Department of Cell Biology, University of Pittsburgh School of Medicine, Pittsburgh, PA, USA

**Keywords:** humanin, aging, age-related disease

## Abstract

Humanin (HN) is 24-amino acid mitochondria-associated peptide. Since its initial discovery over a decade ago, a role for HN has been reported in many biological processes such as apoptosis, cell survival, substrate metabolism, inflammatory response, and response to stressors such as oxidative stress, ischemia, and starvation. HN and its potent analogs have been shown to have beneficial effects in many age-related diseases including Alzheimer’s disease, stroke, diabetes, myocardial ischemia and reperfusion, atherosclerosis, amyotrophic lateral sclerosis, and certain types of cancer both *in vitro* and *in vivo*. More recently, an association between HN levels, growth hormone/insulin-like growth factor-1 (GH/IGF axis), and life span was demonstrated using various mouse models with mutations in the GH/IGF axis. The goal of this review is to summarize the current understanding of the role of HN in aging and age-related diseases.

## Introduction

Humanin (HN) is a novel, 24-amino acid polypeptide with proven effects on cell survival, metabolism, response to stressors, and inflammation *in vivo* and *in vitro*. It was discovered in 2001, using a modified “death-trap” screening, in the unaffected occipital brain of a patient with sporadic Alzheimer’s disease (AD) (1). Since then, it has been identified in a wide range of tissues including testes, colon, hypothalamus, heart, liver, skeletal muscle, kidney, and vascular wall (2–6). Levels of HN are measurable in plasma, cerebrospinal fluid (CSF), and seminal fluid indicating that it is a secreted protein (4, 7–9), though it is still unclear which tissue(s) contributes to the circulating HN pool.

Humanin is believed to be encoded from a small open reading frame (ORF) in the mitochondrial (mt) DNA within the 16S ribosomal RNA coding region. However, there are mitochondrial pseudogenes within the nuclear DNA sequences with great resemblance to HN-encoding small ORF (10). The site of translation of HN has not been fully identified yet and the length of HN molecule would differ based on the site of translation. Because of the differences in translational machinery between the mitochondria and the cytosol, it will be a 21 amino acid peptide if translation occurs in mitochondria, while cytoplasmic translation will yield a 24-amino acid long polypeptide. Both 21 and 24-amino acid peptides are biologically functional proteins (11, 12). A rat cDNA encoding a secreted peptide homologous to HN, named Rattin has also been identified, indicating the existence of HN in other species (13). Indeed, Guo et al. reported that cDNAs identical or similar to the ORF for HN exist in plants, nematodes, rats, mice, and other species (14).

It is hypothesized that HN is a part of the “retrograde signaling” – a vital communication process between mitochondria and nuclear genome that maintains cellular homeostasis and integrity (15). Very few retrograde signaling molecules and pathways have been identified; including Ca^2+^, reactive oxygen species (ROS), nitric oxide (NO), carbon monoxide (CO), and cytochrome *c*; and HN is heralded as the “harbinger” of other mitochondrial-derived peptides by Lee et al. (15).

## Structure of HN

Humanin [Sequence: Met-Ala-Pro-Arg-Gly-Phe-Ser-Cys-Leu-Leu-Leu-Leu-Thr-Ser-Glu-Ile-Asp-Leu-Pro-Val-Lys-Arg-Arg-Ala (MAPRGFSCLLLLTSEIDLPVKRRA)] is encoded from a 75 bp ORF sequence within the 1,567 bp cDNA, which yields either a 21 or 24-amino acid polypeptide depending on the location of translation machinery. By binding to either intra-cellular molecules [such as insulin-like growth factor-binding protein (IGFBP)-3, Bax, Bak, or tBid] (14, 16–19) or membrane receptors (8, 20, 21), HN promotes cell survival in response to a variety of insults, improves insulin sensitivity, increases glucose stimulated insulin secretion (GSIS), as well as prevents oxidative stress-induced damage due to ischemia/reperfusion (I/R), hypoxia, or starvation (refer to related sections below for references).

Humanin has a positively charged N-terminal (Met-Ala-Pro-Arg), central hydrophobic region (Gly-Phe-Ser-Cys-Leu-Leu-Leu-Leu-Thr-Ser-Glu-Ile-Asp-Leu), and negatively charged C-terminal (Pro-Val-Lys-Arg-Arg-Ala) (1, 22). Relatively short structure of HN has enabled researchers to identify the role of each amino acid residue within the polypeptide through systematic single amino acid substitution technique. Last three residues in the C-terminal are accepted as non-essential because both 21 and 24-amino acid long peptides have indistinguishable intracellular and extracellular effects (22).

Yamagishi et al. postulated that the entire HN peptide functions as a signal peptide for extracellular secretion. They demonstrated that the self-secretory function is lost when any of the amino acid domains within the Leu9–Leu11, or Pro19–Val20 structure was substituted for Arg (12). Furthermore, Leu10 was identified as having a central role in this process, because Asp substitution of this amino acid (L10A) but not Leu9 or Leu11 completely abolishes the extracellular secretion (12). With regards to the neuro-protective function, Pro3 to Pro19 is termed as “neuro-protective core domain,” and the amino acids Pro3, Ser7, Cys8, Leu9, Leu12, Thr13, Ser14, and Pro19 within this domain were found to be essential (12). Single amino acid substitution of these positions to Ala to form P3A, S7A, C8A, L9A, L12A, T13A, S14A, and P19A, respectively, completely abrogates the neuro-protective effect of HN molecule, whereas, replacement of Ser14 to Gly (S14G, HNG) results in increased cyto-protective potency of HN over 1,000-fold (23). Replacement of Ser14 with D-form Serine (D-Ser) residue also increases neuro-protective function similar to HNG; whereas, D-Ser7 substitution does not (24).

Ikonen et al. demonstrated that Phe6 and Lys21 are essential sites for binding of HN to its interacting partner, IGFBP-3. While Phe6 to Ala conversion (F6A) completely abolishes the interaction of HN to IGFBP-3, Lys21 to Ala (L21A) conversion blocks the interaction only at lower concentration of IGFBP-3 (16). Combination of the changes at the 6th (F6A) and 14th (S14G) positions creates a more stable, non-IGFBP-3 binding molecule (HNGF6A), which has the ability to modulate insulin action and increase GSIS (25). Recently, Maftei et al. demonstrated that HN (5–15) directly binds to 17–28 region of Amyloid β (Aβ) (1–40) using proteolytic epitope excision and extraction, and affinity-metabolic syndrome (MS) analyses (26). The inhibition of this region of Aβ previously has been shown to effectively decrease aggregation of the neurotoxic amyloid fibrils, and associated cytotoxicity *in vitro* in human neuroblastoma cell line, SH-SY5Y (27).

Nuclear magnetic resonance (NMR) and circular dichroism (CD) studies have shown that the secondary structure of HN and HNG is more disordered in water than in PBS, and the different structure in PBS appears to be due to self-association of the peptide (28). The self-association of HN into dimers and/or oligomers appears to occur and seems to be required for biological activities (12, 24). A complete list of the function of each amino acid and the effect of substitution are summarized in Table 1.

**Table 1:** **Structure of HN peptide and role of individual amino acids**.

Pos.	Amino acid	Function	Effect of amino acid substitution
**N-TERM**			
1	Met (M)		
2	Ala (A)		
**3**	**Pro (P)**	Neuro-protection	Pro3 to Ala (P3A) – abrogates neuro-protective function
**4**	**Arg (R)**		
**HYDROPHOBIC CORE REGION**			
**5**	**Gly (G)**		
**6**	**Phe (F)**	IGFBP-3 binding, Aβ binding	Phe6 to Ala (F6A) – abrogates IGFBP-3 binding
**7**	**Ser (S)**	Aβ protection	Ser7 to Ala (S7A) – abrogates cyto-protective and neuro-protective functions, and prevents dimerization
		Aβ binding	
		Dimerization	
**8**	**Cys (C)**	Neuro-protection	Cys8 to Ala (C8A) – abrogates neuro-protective function
		BAX, BAD, and tBID binding	
**9**	**Leu (L)**	Neuro-protection	Leu9 to Arg (L9R) – Non-secretory, but retains function when added into the medium
		Secretion	
		Dimerization	Leu9 Ala (L9A) – prevents dimerization and abrogates neuro-protective function
**10**	**Leu (L)**	Secretion	Leu10 to Asp (L10D) – abrogates secretion
			Leu10 to Arg (L10R) – abrogates secretion
**11**	**Leu (L)**	Secretion	Leu11 to Arg (L11R) – abrogates secretion
**12**	**Leu (L)**	Neuro-protection	Leu12 to Ala (L12A) – abrogates neuro-protective function
**13**	**Thr (T)**	Neuro-protection	Thr13 to Ala (T13A) – abrogates neuro-protective function
**14**	**Ser (S)**	Neuro-protection	Ser14 to Gly (S14G) increases cyto-protective potency over 1,000-fold. This isoform is active when monomeric
			Ser14 to D-Ser increases potency
			Ser14 to Ala (S14A) – abrogates neuro-protective function
**15**	**Glu (E)**		
**16**	**Ile (I)**		
**17**	**Asp (D)**		
**18**	**Leu (L)**		
**C-TERM**			
**19**	**Pro (P)**	Aβ protection	Pro19 to Ala (P19A) – abrogates neuro-protective function
		Secretion	Pro19 to Arg (P19R) – abrogates secretion
20	Val (V)	Secretion	Val20 to Arg (V20A) – abrogates secretion
21	Lys (K)	IGFBP-3 binding	Lys21 to Ala (L21A) – blocks interaction at lower IGFBP-3 concentrations
22	Arg (R)	?Non-essential	
23	Arg (R)	?Non-essential	
24	Ala (A)	?Non-essential	

*Bolded: neuro-protective core domain (NPCD)*.

Benaki et al. investigated the structure of synthetic HN in aqueous and 30% 2,2,2-trifluoroethanol (TFE) solutions and reported that in aqueous solution, HN exists predominantly in an unstructured conformation in equilibrium with turn-like structures involving residues Gly5 to Leu10 and Glu15 to Leu18, providing indication of nascent helix (29). In the less polar environment of 30% TFE, HN readily adopts helical structure with long-range order spanning residues Gly5 to Leu18 (29). Comparative 3D modeling studies and topology predictions also support the observation. These studies reveal the flexibility of HN molecule in aqueous environment, which makes it free to interact with possible receptors that mediate its action, but also the ability of HN to acquire a helical conformation necessary for specific interactions and/or passage through membranes (29). Another study showed that HN is readily stabilized in an ordered helical conformation in the TFE/water mixture, but kept partly unfolded in water (30).

## HN Signaling Pathways

**Figure 1:**
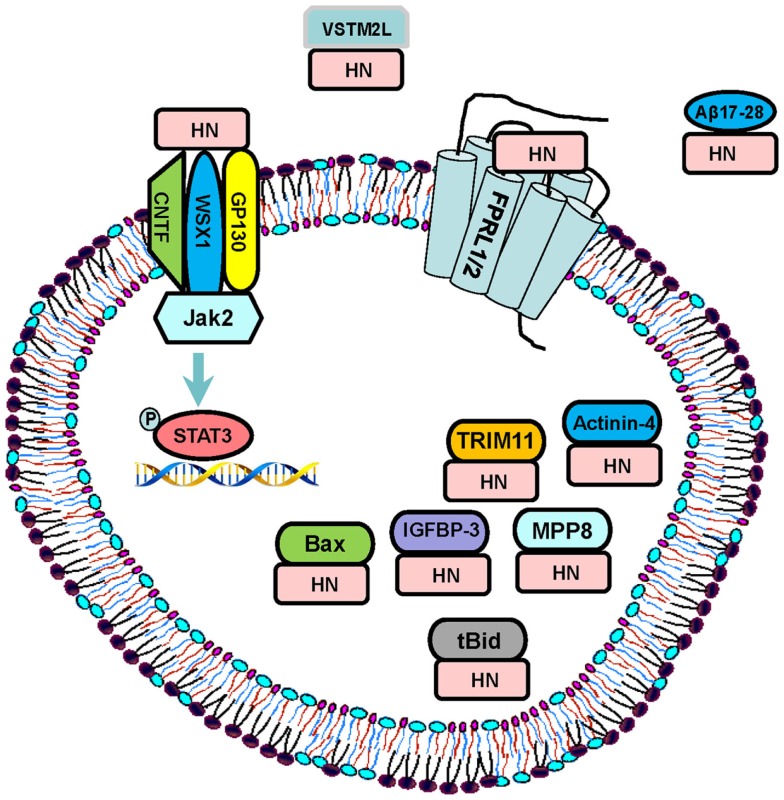
**Signaling pathways: HN exerts its function through both extracellular receptors and intracellular binding partners**. The proposed extracellular receptors are trimeric receptor complex including CNTF, WSX1, and GP130 and downstream JAK2–STAT-3 signaling pathway, and FPRL-1/2 G-protein-coupled receptors. HN directly binds to Aβ 17–28 and prevents the interaction of Aβ42 with receptors. HN also binds to its intracellular partners including IGFBP-3, Bax, tBid, MPP8, TRIM11, and actinin-4. VSTM2L is an extracellular antagonist of HN.

Humanin exerts its diverse functions through binding to both intracellular molecules and putative cell membrane receptors (Figure 1). Guo et al. demonstrated that HN binds to Bcl-2-associated X protein (Bax), a pro-apoptotic signaling peptide. Utilizing yeast two-hybrid co-immunoprecipitation studies, the authors demonstrated that wild type HN specifically binds to inactive form of Bax, inhibits the conformational changes and transfer of Bax from cytosol to the mitochondria, and thereby suppressing cytochrome *c* release, an initiation step of apoptosis, and inhibiting staurosporine-induced death (14). Another intracellular pro-apoptotic protein “BH3 interacting-domain death agonist” (Bid), and its truncated form (tBid) were also shown to bind to HN and contribute to its anti-apoptotic effect (19). Activation of Bid involves proteolytic removal of its N-terminal and myristoylation to form tBid, which then translocates to the mitochondria, leading to the mitochondrial permeabilization and cytochrome *c* release (31). HN does not block proteolysis of Bid nor prevents the translocation of tBid to mitochondria, but rather inhibits the association of tBid with Bax and as a consequence, prevents tBid-induced oligomerization of Bax, and suppresses release of cytochrome *c*. Furthermore, Ikonen et al. demonstrated that HN binds to and modulates the pro-apoptotic function of IGFBP-3, and regulates cell survival (16). Other intracellular molecules that have been shown to bind to HN are actinin-4 (32), a tripartite motif protein TRIM11 (33), and M-phase phosphoprotein 8 (MPP8) (Figure 1) (34). Recently, a V-set and transmembrane domain containing two like (VSTM2L) protein, was demonstrated to co-localize with HN in distinct brain areas as well as in primary cultured neurons, and function as an antagonist of HN (Figure 1) (35).

In terms of the extracellular signaling by HN, Ying et al. reported that HN induces chemotaxis of human mononuclear phagocytes by binding to human G protein-coupled formyl peptide receptor-like-1 (FPRL-1) and its murine counterpart FPRL-2 (20). Interestingly, FPRL-1 and FPRL-2 are also functional receptors for Amyloid β (Aβ) 42, an important peptide in the pathophysiology of AD-related neuronal toxicity, suggesting that HN may exert its neuro-protective effects also by competitively inhibiting the access of FPRL-1 to Aβ 42 (20). This hypothesis was further supported by Harada et al. who demonstrated that HN directly binds to FPRL-1 and 2 receptors in Chinese hamster ovary cells expressing the corresponding receptors after transfection (21). On the contrary, Hashimoto et al. showed that HN exerts neuro-protection against Aβ and activates signal transducer and activator of transcription (STAT) 3 in F11 cells even after siRNA-mediated disruption of FPR2, the mouse counterpart of FPRL-1 (36), suggesting the existence of alternative receptor(s) of HN. In fact, they discovered a tripartite cytokine-like receptor complex [belonging to the interleukin (IL)-6 receptor family] comprising the ciliary neurotrophic factor (CNTF) receptor, the IL-27 receptor WSX1, and glycoprotein (gp)130, activation of which upregulates the Janus Kinase (JAK) 2 and STAT-3 pathways (8). Based on these findings, it is speculated that different cell types express different cell membrane receptors to respond to HN (11). The signaling pathways and binding partners of HN are summarized in Figure 1.

Humanin has been shown to activate other signaling pathways besides JAK2–STAT-3, including p38 mitogen-activated protein kinases (p38MAPK) (in human K562 cells, primary rat neurons, and mouse germ cells *in vivo*) (37, 38), AMP-activated protein kinase (AMPK) (mouse cardiac I/R model *in vivo*) (3), insulin signaling *in vivo* in mouse models (39–41), and ERK1/2 *in vitro* (20). It is also reported that HN inhibits amyloid precursor protein (APP) induced c-Jun N-terminal kinase (JNK) activation and thereby protects neuronal cells from apoptosis (42).

## Regulation of HN

The endogenous regulation of HN under physiological conditions is not yet established. We have shown that the circulating levels of HN decrease with age in both human and mice (43). A relationship between HN and GH/IGF axis was reported in a recent publication. Long-lived, GH-deficient Ames mice (that have decreased GH and IGF-1) displayed elevated HN levels, while short-lived GH-transgenic mice (high GH and high IGF-1) have reduced HN levels. This relationship was further confirmed in mice and humans; treatment with GH or IGF-1 reduced circulating HN levels (44). Using LID mice (liver specific IGF-1 deletion model-where the GH levels are high, while IGF-1 levels are low) and IGFBP-3 knock-out mice (where free IGF-1 levels are high), the authors demonstrated that the levels of HN are inversely proportional to circulating IGF-1 levels. There is increased expression of HN in response to GH and IGF-I in cultured rat Leydig cells between 10 and 40 days of life but not at 2 months of age (6) suggesting a developmental regulation. Niikura et al. identified that HN interacts with a putative E3 ubiquitin ligase TRIM11, which leads to the degradation of HN through the proteasome pathway, and regulation of the intracellular level of *de novo* synthesized HN (33).

Endogenous up-regulation of HN has been demonstrated in certain pathological states. It is shown that HN peptide level is strongly increased in mitochondria and siderosomes in diffuse-type of pigmented villonodular synovitis (45). Increase in HN expression is also demonstrated in the muscles of patients with chronic progressive external ophthalmoplegia compared to those in control fibers (5). An increased HN expression level in skeletal muscles from patients with mitochondrial encephalomyopathy with lactic acidosis and stroke-like episodes (MELAS) was also reported (46). The authors suggested that the increase in HN could be an initial compensation for the defects in energy production in the affected muscle fibers; while further progressive defects may ultimately lead to degenerative ragged-red fibers. Our findings of increased intracellular levels of HN after myocardial ischemia and reperfusion (MI-R) and persistence as long as 24 h in mouse cardiac tissue (3) also suggest a compensatory up-regulation of HN in stress conditions.

## Role of Humanin in Age-Related Diseases

The discovery of HN molecule and the demonstration of its neuro-protective activity in AD inspired researchers from different fields to study the potential role of HN in the pathophysiology of other neurological and non-neurological diseases (1). To date, HN and its analogs have been demonstrated to play a role in multiple diseases including type 2 diabetes (25, 43), cardiovascular disease (CVD) (2, 3, 47), memory loss (48), amyotrophic lateral sclerosis (ALS) (49), stroke (50), and inflammation (22, 51). The mechanisms that are common to many of these age-related diseases are oxidative stress (52) and mitochondrial dysfunction (53). Mitochondria are major source of ROS, excess of which can cause oxidative damage of cellular lipids, proteins, and DNA. The accumulation of oxidative damage will result in decline of mitochondrial function, which in turn leads to enhanced ROS production (53). This vicious cycle can play a role in cellular damage, apoptosis, and cellular senescence – contributing to aging and age-related diseases. Indeed, oxidative stress is tightly linked to multiple human diseases such as Parkinson’s disease (PD) (54), AD (55), atherosclerosis (56), heart failure (57), myocardial infarction (58), chronic inflammation (59), kidney disease (60), stroke (61), cancers (62, 63), and many types of metabolic disorders (64, 65). We and others have shown that HN plays critical roles in reducing oxidative stress (66–68). In this section, we will summarize the current knowledge on the role of HN in various age-related diseases.

## HN and Neurological Diseases

### Alzheimer’s disease

Alzheimer’s disease is predicted to affect 1 in 85 people globally by 2050. Short-term memory loss and impairment of other cognitive domains are clinical hallmarks of the disease; whereas, cerebral cortical atrophy due to neuronal cell loss particularly in the temporal–parietal lobes, hippocampus and to a lesser extent in the frontal lobes, accumulation of extracellular senile plaques made of amyloid-beta (Aβ) protein (cleavage product of APP), and hyper-phosphorylation of the microtubule associated tau-protein are the main imaging and pathophysiological markers (25, 50). The mechanism of the Aβ induced neuronal cell death has not yet fully elucidated; however, multiple groups have postulated the presence of death receptors as the potential mechanism of such insult [for review see Ref. (27)].

Role in AD has been on the main focus of HN-related research. The effects of HN on AD-related pathology as well as functional correlates have been tested in *in vitro* systems and in *in vivo* mouse models. Hashimoto et al. demonstrated that HN suppresses neuronal cell death induced by Aβ and three different types of familial AD (FAD) genes including mutant APP, presenilin (PS)1, and PS2 (1). In addition, HN inhibits neurotoxicity by AD-relevant insults induced by other FAD genes including A617G-APP, L648P-APP, A246E-PS1, L286V-PS1, C410Y-PS1, and H163R-PS1 and other Aβ peptides (Aβ 1–42 and Aβ 25–35) (1). They showed that HN mediated suppression of the neuronal cell death induced by K595N/M596L-APP (NL-APP) is not through the inhibition of Aβ1-42 secretion but via the HN’s inhibitory action on intracellular toxicity triggered by NL-APP and Aβ (69). Furthermore, they showed that secretion is required for the neuro-protective effects by using non-secreted biologically active point mutant HN analog (L9R), which is retained in the cytoplasm and did not exert any cyto-protection but was protective when added to the culture medium (1). Tsukamoto et al. demonstrated that Aβ toxicity in the neuronal cell line can be completely suppressed with HN via one of the proposed death receptor, the 75-kDa neurotrophin receptor (p75 NTR) (47–49). HN protects PC12 neuronal cells from Aβ-induced viability loss and cell apoptosis, decreases mitochondrial membrane potential and prevents cytochrome *c* release from mitochondria-key steps in apoptosis (70). A potent HN analog, HNG, has been shown to not only inhibit the formation of the Aβ1–42 fibrils but also cause disaggregation of the preformed fibrils, which provides novel evidence that HNG may have anti-Aβ aggregation and anti-fibrillogenesis, as well as fibril-destabilizing properties (71).

With intra-cerebro-ventricular (ICV) injection of HNG, Aβ-induced impairment of short-term/spatial working memory was prevented *in vivo* (72). Interestingly, intraperitoneal (IP) administration of HNG also ameliorated behavioral deficits, and reduced neuro-inflammatory responses and apoptosis induced by ICV injection of aggregated Aβ 25–35 in mouse brain (73). In a subsequent study, the group showed that chronic treatment (3-month IP injection) of HNG: (i) significantly improves spatial learning and memory deficits, (ii) reduces Aβ plaque accumulation and insoluble Aβ concentrations, and (iii) decreases neuro-inflammatory responses in middle-age APPswe/PS1dE9 mice, a double transgenic mouse model of AD over expressing APP, and mutant human PS-1 in neurons. Similar pathological and functional improvements were also demonstrated in APPswe, tauP310L, and PS-1M146V triple transgenic mice following treatment with HNG. Interestingly, in this model, though there was reduced Aβ accumulation, no changes in tau phosphorylation levels were noted (74). This suggests that the cyto-protective effect of HN is independent of the phosphorylation and aggregation of tau-protein. However, more direct and detailed studies are needed to characterize any potential effect of HN on tau-protein aggregation induced memory loss and learning deficit (75). Novel HN delivery system, using a transducible HN with an extended caspase-3 cleavage sequence (tHN-C3), was shown to protect neurons against H_2_O_2_ and soluble Aβ42 induced cell death *in vitro* through binding to Bax. Delivering HN through this system was also found to decrease inflammatory cell infiltration, decrease apoptosis in neurons and improve memory learning deficits in genetic (Tg2576) and Aβ42 induced AD mouse models, and protect rats from I/R induced brain injury (76).

Currently, there is no definitive cure for AD (31). The multiple *in vitro* and *in vivo* studies showing that HN or its potent analogs protect from AD-related neuronal cell death and functional impairments, offer significant promise for a potential role for HN as a new treatment approach to treat AD.

### Stroke

Stroke [cerebrovascular accident (CVA)] was the second leading cause of death worldwide according to World Health Organization (WHO) report in 2012 (http://who.int/mediacentre/factsheets/fs310/en/). CVA can be either ischemic or hemorrhagic; ischemic subtype constitutes roughly 80% of the all strokes (77). The role of HN has been studied in both ischemic and hemorrhagic types of stroke in mouse models *in vivo*.

Humanin either alone or in combination with other neuro-protective factors has been shown to offer protection in cerebral I/R mice models *in vivo*. Xu et al. demonstrated that ICV infusion of HNG resulted in smaller infarct volume, decreased number of apoptotic neurons, and improved neurological function in middle cerebral artery occlusion induced I/R injury in mice (50). This was associated with inhibition of ERK (a member of MAPK signaling pathway) phosphorylation and poly (ADP-ribose) polymerase (PARP) activity, a marker of caspase-3 activity (50). Same group also showed that activation of PI3 kinase/Akt signaling pathway is important in HNG’s neuro-protective function (40). Furthermore, they also showed that combination of treatment with HNG and a necroptosis inhibitor necrostatin-1 (Nec-1) results in more robust neuro-protection than HNG or Nec-1 alone on I/R induced cerebral infarct (78). The synergistic effects of HNG and Nec-1 on hypoxia induced neuronal cell death were also replicated in *in vitro* experiments using cultured mouse primary cortical neurons (78). Others reported that HN protects cortical neurons from I/R injury through an increase in activity of superoxide dismutase (SOD) (79). Wang et al. showed protective roles of HNG in a mouse model of intracranial hemorrhage following IP administration within 1 hour post injury (41); treatment with HNG resulted in decreased brain edema, neuronal cell death, and injury volume while improving neurological recovery.

The results of the limited studies are promising; however, more studies are needed to better understand if HN can be used as a therapeutic agent in the management of CVA.

### Other neurological conditions

Humanin was shown to play role in certain other neurological diseases. HN was found to offer protective effect from Prion-peptide (PrP) (118–135)-induced cell death, but not against PrP (106–126). The reason for this selective protection is not clear though the mechanistic differences between these two PrPs inducing apoptosis could be a contributor (80). Mamiya et al. evaluated the effects of the scopolamine-HBr induced impairment of spontaneous alternation behavior in mice using the Y-maze as an index of short-term memory, and found that HNG reversed the anti-cholinergic drug mediated impairment of the learning and memory function in mice (48). In a recent publication, Cui et al. reported that HN rescued cortical neurons from excitatory toxicity caused by NMDA in a dose-dependent manner without interacting with the receptors, and pointed to the potential role of its use in preventing the damage caused by this pathway (81). However, studies have shown that HN failed to protect against certain cytotoxicity including Q79, SOD mutants, etoposide, Fas, or basally occurring death indicating specificity in action (1).

## HN and Cardiovascular Diseases

Cardiovascular disease is the number one leading cause of death in the United States and worldwide (82). Aging is the biggest risk factor for the development of CVDs; risk approximately triples with each decade of life (83). American Heart Association statistics report in 2006 states that 88% of people who died of coronary heart disease were 65 and older (84).

We and others have shown that heart expresses the highest level of HN at both mRNA and protein levels (3, 13, 67). HN is expressed in the endothelial cell lining of the coronary arteries, human internal mammary arteries, and sections of the greater saphenous vein as demonstrated by immune-staining (67). Endogenous HN levels increase after myocardial I/R in mice, and HN is present in the atherosclerotic plaques of the coronary arteries suggesting a role for HN in cardiovascular health.

When HNG is administered exogenously, either pre-ischemia or at the time of reperfusion in a mouse model of myocardial I/R, there is a dose-dependent decrease in infarct size (3). In addition, HNG treated mice demonstrated better cardiac function post MI-R as demonstrated by improved ejection fraction, end-systolic volume and end-diastolic volume, and cardiac output (3). Furthermore, we also observed that HNG protects cardiac myoblasts from oxidative stress-induced cell death by acutely increasing activity of antioxidants through involvement of non-receptor tyrosine kinases (66).

Widmer et al. reported that human coronary endothelial function dysfunction is associated with lower systemic HN levels, introducing a potential diagnostic and/or therapeutic target for patients with coronary endothelial dysfunction (9). The same group also demonstrated that the treatment with exogenous HN protected endothelial cell cultures from Ox-LDL-induced oxidative stress and apoptosis (67). Daily IP injection of HNGF6A for 16 weeks prevented endothelial dysfunction and decreased atherosclerotic plaque size in the proximal aorta of Apo-E deficient mice fed with a high-cholesterol diet, attributable to the reduction in apoptosis rate and preservation of eNOS activity (68). In the same mouse model, it was shown that HN attenuates renal microvascular remodeling, inflammation, and apoptosis in the early stage of kidney disease, indicating that HN may serve as a novel therapeutic target to mitigate kidney damage in early atherosclerosis (2). HN was also demonstrated in the carotid atherosclerotic plaques in humans, and the expression level of HN was found to be inversely correlated with the stability of the plaques, i.e., more HN was present in unstable plaques and patients from stroke subgroup (47). Whether this suggests a direct role of HN in the formation or stability of the carotid atherosclerotic plaques needs to be established.

## HN and Metabolic Effects

T2DM is one of the most common metabolic diseases and its prevalence directly correlates with increasing age, peaking at 60–74. Almost one-third of the elderly have diabetes and three quarters have diabetes or pre-diabetes (85–87). Age associated changes in hormonal milieu along with changes in body composition contribute to insulin resistance and increased incidence of diabetes. In addition, aging is associated with decreased beta-cell proliferative capacity and enhanced sensitivity to apoptosis (88). Both peripheral insulin resistance and impaired insulin secretion contribute to the pathogenesis of T2DM in aging (86, 89).

Role of HN in glucose homeostasis has been studied using hyperinsulinemic-euglycemic clamp and hyperglycemic clamp techniques. Hyperinsulinemic-euglycemic clamp is the gold standard method to assess *in vivo* insulin sensitivity and allows assessment of insulin sensitivity specifically at the level of liver and muscle. During hyperinsulinemic-euglycemic clamps, rats receiving continuous ICV infusion of HN required higher glucose infusion rate (GIR) to maintain normoglycemia as a result of decreased hepatic glucose output and increased glucose uptake in the skeletal muscle, demonstrating increased insulin sensitivity. The central action of HN was shown to be mediated via activation of hypothalamic STAT-3 signaling pathway (43). Our group also demonstrated that continuous IV infusion of HNGF6A, HN analog that is stable, potent, and non-IGFBP-3 binding, during hyperinsulinemic-euglycemic clamp increased GIR, peripheral glucose uptake, and suppressed hepatic glucose production. Moreover, HNGF6A, when given as a single IV injection, significantly lowered the blood glucose in Zucker diabetic fatty rats (43). In addition, we showed that HN increases glucose uptake into the β cells, enhances glucose oxidation resulting in an increased GSIS as demonstrated *in vivo*, in cultured beta cells and in islets isolated from wild type and diabetic mice (25). These studies indicate a role for HN in whole body glucose homeostasis through both improved insulin action and increased insulin secretion.

Non-obese diabetic mouse is an autoimmune model for T1DM; the development of diabetes is time-dependent as age correlates with increased lymphocyte infiltration, decreased beta-cell proliferation, and enhanced sensitivity to glucose-induced β-cell apoptosis (88). Treating non-obese diabetic (NOD) mice with HN for 6 weeks normalized glucose tolerance and treatment for 20 weeks prevented/delayed the onset of diabetes in these mice, secondary to decreased lymphocyte infiltration in the islets and decreased apoptosis.

Humanin expression is increased in small arteries along with succinate dehydrogenase positive staining in MELAS muscle fibers, and synthesized HN increases cellular ATP levels by directly acting on mitochondria in TE671, a human rhabdomyosarcoma cell line (46). We also reported that HNGF6A increases mitochondria metabolism and ATP generation in the cultured β cells (25) showing a role for HN in substrate metabolism.

The prevalence of MS also increases with age (90). MS, polycystic ovarian syndrome, non-alcoholic fatty liver disease, and dyslipidemia are closely associated with insulin resistance (91, 92). A future direction would be to determine whether HN has a role in the pathophysiology of these diseases.

## HN and Inflammation

Obesity, CVD, diabetes, chronic kidney disease, and AD are associated with a chronic inflammatory state (87, 93–95). Aging *per se* is characterized by chronic low-grade systemic inflammation even in the absence of chronic disease, as the circulating levels of pro-inflammatory cytokines such as IL-6, tumor necrosis factor (TNF)-α, and acute phase proteins such as C-reactive protein (CRP) and serum amyloid A (SAA) increase by two to fourfold in elderly (96). Chronic inflammation in the elderly may contribute to multiple diseases, poor physical functioning, and mortality (97). Therefore, reversing the process of chronic inflammation or, at least, slowing it down has been an attractive area of research in an effort to prevent or delay morbidities/mortalities associated with these conditions.

A role for HN in down-regulation of inflammatory responses has been demonstrated *in vivo* and in cell culture systems. Miao et al. first observed that HNG ameliorates Aβ25–35-induced neuro-inflammatory responses by decreasing the level of IL-6 and TNF-α in mice (73). This important finding may suggest that in addition to exerting functions through binding to membrane receptors and intra-cellular molecules, HNG also offers neuro-protection in AD by altering the inflammatory response. Zhang and colleagues discovered that HN attenuates inflammation by down-regulating intra-renal inflammatory markers of MCP-1, TNF-α, and osteopontin and reduces macrophage infiltration in hypercholesterolemic Apo-E deficient mice; thereby decreasing the renal microvascular remodeling, inflammation, and apoptosis in the early stage of kidney disease (2). Finally, Zhao et al. reported that treatment of HNG partially suppresses the secretion of pro-inflammatory cytokines including IL-6, IL-1β, and TNF-α in a dose-dependent manner in astrocytes induced by lipopolysaccharides (LPS) (51).

## HN and Cancers

Age-related increase in cancer risk, with leveling off at advanced ages is well-established (98). HN’s well-known anti-apoptotic property raised concern among the researchers whether HN contributes to cancer development and helps cancer cell survival. Maximov et al. hypothesized that HN is an oncopeptide (99), based on the finding of up-regulation of expression of the mitochondrial 16S rRNA gene in non-Hodgkin’s lymphoma, which potentially enhances HN expression. Furthermore, HN was immunologically detected in the serum of some patients with cutaneous T-cell lymphoma but not in healthy subjects (100). Using the yeast two-hybrid model, the Maximov group identified MPP8 as a binding partner for HN (101), which is a previously described oncoprotein playing a role in tumor motility and invasion (101); and thus pointing to the potential role of HN in oncogenesis. Recently, a suppression subtractive hybridization study showed the over-expression of HN and isoforms in chemo-resistant tumor gastric tissues from patients. Utilizing qRT-PCR, the authors confirmed the increased mRNA levels of HN1, HN3, HN6, and HN10 genes, suggesting potential role of HN in the development of chemo-resistance in gastric tumor cells. The limitation of the study, as noted by the authors, was the lack of protein level measurements that could not be performed due to lack of isoform-specific antibodies.

Contrary to the potential role of HN in tumorigenesis and metastasis of the cancer cells, Eriksson and colleagues demonstrated that, when HNG is administered with bortezomib [proteasome inhibitor currently studied in clinical trials of childhood leukemia and other cancers that induces apoptosis in growth plate chondrocytes and impair linear bone growth in treated mice (102–107)], HNG prevented bortezomib-induced bone growth impairment in mice with human tumor xenograft models (108), without affecting its chemotherapeutic effects. They also showed in human tumor xenograft and cell lines that HNG prevented bortezomib-induced apoptosis by preventing Bax and PARP activation (108). More interestingly, HNG alone delayed tumor growth and tumor doubling time in cancers such as medulloblastoma and neuroblastoma *in vivo* (108). The authors speculated that increased Bax in chondrocytes compared to human neuroblastoma cells could be the basis for chondrocyte-rescuing effect of HNG following bortezomib treatment.

The current data on the relationship between HN, tumorigenesis, and cancer metastasis are not conclusive. More studies are needed to understand if the changes in expression of HN are tumor-type specific, whether they are a cause or a compensatory response and if they affect the response to treatment.

## Perspective

Since its discovery, HN has been demonstrated to offer beneficial effects in many diseases, many of which are age-related. The observation that levels of HN decline with age further supports the role of HN in aging and age-related diseases. Diseases which were earlier considered age-related such as T2DM are now seen in much younger ages due to the burgeoning epidemic of obesity. The role of HN in mitigating the effects of bortezomib on growth plate chondrocyte apoptosis and islet cell apoptosis on autoimmune mouse model of diabetes extend the potential role of HN beyond the spectrum of age-related diseases. HN and analogs offer promise as a potential therapeutic option for neurodegenerative disorders, CVDs, diabetes, and a potential adjunct to chemotherapy. Further studies are needed to evaluate the pharmacokinetics and safety profile for long term use. Gain and loss of function models are necessary to fill the gaps in our current knowledge and enhance our understanding of the physiological role of this peptide and its role in various diseases.

## Conflict of Interest Statement

This work is supported by National Institute of Health Grant R-01 AG035114 to Radhika Muzumdar. Dr. Radhika Muzumdar is an inventor on patents and patent applications covering the use of humanin and humanin analogs for the treatment of diabesity, myocardial infarction, and insulin secretion. Some of these patents and patent applications have been licensed to a startup company in which she has a financial interest. The other co-authors declare that the research was conducted in the absence of any commercial or financial relationships that could be construed as a potential conflict of interest.
